# Supplementary material for: Analysis of the Response of Chlamydomonas reinhardtii to Cobalt Ions Reveals the Protective Role of Thiols, Ascorbate, and Prenyllipid Antioxidants, and the Negative Impact of Cobalt Toxicity on Photoprotective Mechanisms
Source: Plants (Basel). 2025 Nov 16;14(22):3496. doi: 10.3390/plants14223496 (PMC12656169; doi:10.3390/plants14223496)
Supplement: Supplementary file 1 [file plants-14-03496-s001.zip › plants-3893767-supplementary.pdf]

Analysis of the response of *Chlamydomonas reinhardtii* to cobalt ions reveals the protective role of thiols, ascorbate and prenyllipid antioxidants, and the negative impact of cobalt toxicity on photoprotective mechanisms

Aylin Kökten and Beatrycze Nowicka

Supplementary file

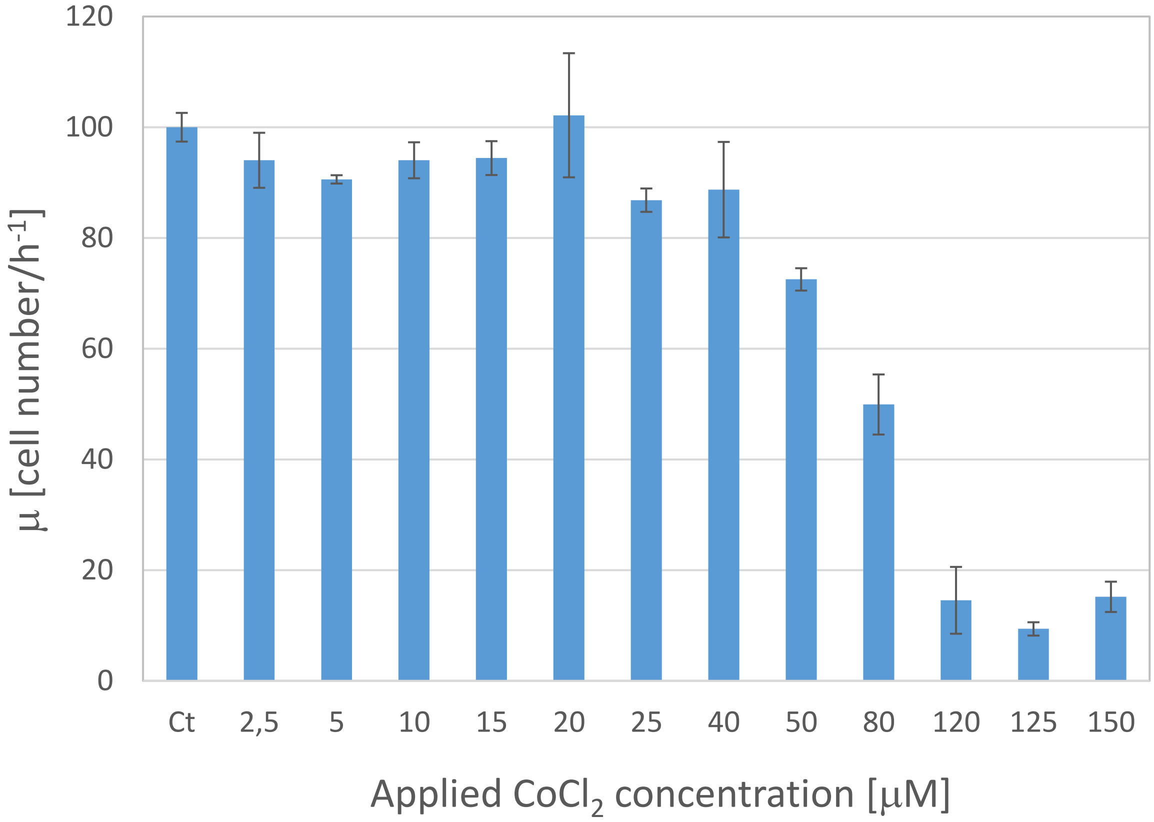

Supplementary Figure 1. Growth rate of $CoCl_2$-exposed *Chlamydomonas reinhardtii* cultures, normalized to control series (assumed as 100%), measured during preliminary experiments. Data are means ± SD (n = 3).
